# Evaluation of *Burkholderia mallei* Δ*tonB* Δ*hcp1* (CLH001) as a live attenuated vaccine in murine models of glanders and melioidosis

**DOI:** 10.1371/journal.pntd.0007578

**Published:** 2019-07-15

**Authors:** Nittaya Khakhum, Preeti Bharaj, Julia N. Myers, Daniel Tapia, David H. Walker, Janice J. Endsley, Alfredo G. Torres

**Affiliations:** 1 Department of Microbiology and Immunology, University of Texas Medical Branch, Galveston, Texas, United States of America; 2 Department of Pathology, University of Texas Medical Branch, Galveston, Texas, United States of America; Fort Collins, UNITED STATES

## Abstract

**Background:**

Glanders caused by *Burkholderia mallei* is a re-emerging zoonotic disease affecting solipeds and humans. Furthermore, *B*. *mallei* is genetically related to *B*. *pseudomallei*, which is the causative agent of melioidosis. Both facultative intracellular bacteria are classified as tier 1 select biothreat agents. Our previous study with a *B*. *mallei* Δ*tonB* Δ*hcp1* (CLH001) live-attenuated vaccine demonstrated that it is attenuated, safe and protective against *B*. *mallei* wild-type strains in the susceptible BALB/c mouse model.

**Methodology/Principal finding:**

In our current work, we evaluated the protective efficacy of CLH001 against glanders and melioidosis in the more disease-resistant C57BL/6 mouse strain. The humoral as well as cellular immune responses were also examined. We found that CLH001-immunized mice showed 100% survival against intranasal and aerosol challenge with *B*. *mallei* ATCC 23344. Moreover, this vaccine also afforded significant cross-protection against *B*. *pseudomallei* K96243, with low level bacterial burden detected in organs. Immunization with a prime and boost regimen of CLH001 induced significantly greater levels of total and subclasses of IgG, and generated antigen-specific splenocyte production of IFN-γ and IL-17A. Interestingly, protection induced by CLH001 is primarily dependent on humoral immunity, while CD4^+^ and CD8^+^ T cells played a less critical protective role.

**Conclusions/Significance:**

Our data indicate that CLH001 serves as an effective live attenuated vaccine to prevent glanders and melioidosis. The quantity and quality of antibody responses as well as improving cell-mediated immune responses following vaccination need to be further investigated prior to advancement to preclinical studies.

## Author summary

Glanders (caused by *Burkholderia*. *mallei*) and melioidosis (caused by *B*. *pseudomallei*) are severe infectious diseases of concern worldwide because of the rising number of cases and mortality rate. The low infectious doses of these two pathogens along with their amenability for aerosolization are factors that could be exploited as potential biothreat agents. Once the diseases have developed in humans and animals, intrinsic resistance to broad classes of antibiotics becomes a challenge for treatment and increases the risk for relapse. The progress in vaccine development demonstrates that live attenuated vaccine strains are the most effective in protection and providing long-lasting immune responses against both diseases. Our data indicate that the *B*. *mallei* double mutant (Δ*tonB* Δ*hcp1*) strain CLH001, is a feasible vaccine candidate to prevent glanders and melioidosis, especially for biodefense and public health purposes.

## Introduction

*Burkholderia mallei* is an aerobic Gram-negative bacillus, an obligate mammalian pathogen, and the causative agent of glanders [1]. This bacterial infection primarily affects horses but also mules, donkeys, goats, dogs, and cats [2]. Humans are an accidental host of *B*. *mallei* infection, either through cutaneous inoculation or inhalation but most often by direct contact with infected animals, carcasses, or laboratory exposure [3,4,5]. Recent outbreaks of glanders in horses have occurred in diverse endemic areas located in Asia, Africa, and South America [1,6]. *B*. *mallei* has been classified as a re-emerging pathogen and as an increasing risk for human infections [5]. Evidence from DNA sequence and 16S rRNA sequence comparisons demonstrates that *B*. *mallei* has evolved by reduction evolution as a clonal derivative of *B*. *pseudomallei* [7]. *B*. *pseudomallei* is also a Gram-negative encapsulated bacillus found in the soil and water of endemic areas and causative agent of a severe and often life-threatening infectious disease known as melioidosis [8,9]. The infections caused by *B*. *mallei* and *B*. *pseudomallei* range from acute to chronic infections. Relapse is also common and occurs after long-term antibiotic treatment [9]. Both *B*. *mallei* and *B*. *pseudomallei* are classified as Tier 1 Select Agents by the Centers for Disease Control and Prevention (CDC) due to their potential use as biothreat agents [10]. The high mortality rate linked to these diseases results from delays in proper treatment and challenge for clinical identification and laboratory diagnostics. Therefore, an effective vaccine against glanders and melioidosis is urgently needed for disease prevention.

Presently, there is no approved vaccine against *B*. *mallei* and *B*. *pseudomallei* infections; however, *Burkholderia* live-attenuated vaccines have been shown as the most effective candidates inducing rapid and broad protective immunity [11,12,13,14,15,16,17,18,19]. Our laboratory previously constructed a candidate live attenuated vaccine based on a single deletion of the iron acquisition energizer gene *tonB*, in either *B*. *mallei* (TMM001) [16] or *B*. *cenocepacia* (GPA001) [18]. The lack of the *tonB* gene in these *Burkholderia* strains resulted in attenuated characteristics; however, these mutant strains presented some safety issues. For example, the iron-dependent phenotype of the TMM001 and GAP001 strains can revert by iron supplementation to partially restore the virulence phenotype. Therefore, a double mutant in a gene encoding a type 6 secretory system (T6SS) structural protein, *hcp1*, was created in *B*. *mallei* (CLH001) [17] and the double mutant re-created in *B*. *pseudomallei* (PBK001) [19]. These double mutant strains are attenuated and provide full protection against wild-type strain infections, while the lack of persistence in the host tissue further increases safety of these vaccine strains [17,19].

The immunogenicity and protective capacity of the *B*. *mallei* CLH001 vaccine were also previously characterized against acute respiratory glanders. The intranasal (i.n.) administration of the CLH001 vaccine in BALB/c mice showed remarkable attenuated characteristics without detectable levels of bacteria or organ pathology [17]. Moreover, vaccination with three dose regimens of 1.5 x 10^5^ CFU of CLH001 vaccine provided full protection of BALB/c mice against a lethal dose (1.5 x 10^4^ CFU) of *B*. *mallei lux* (CSM001) strain and 87.5% survival against a higher dose (3.5 x 10^5^ CFU) of *B*. *mallei* ATCC 23344 using an i.n. challenge. Further, CLH001 was the first *B*. *mallei* strain to be excluded from the restrictions of the U.S. Federal Select Agent Program due to strong data supporting full safety and absence of wild-type reversion [17].

Most glanders and melioidosis vaccine candidates developed today have been tested using two common mouse strains, BALB/c and C57BL/6 [20,21,22]. BALB/c (Th2-prototypical type strain) represents a susceptible mouse that is used for mimicking acute disease, while C57BL/6 mice (Th1-prototypical type strain) are more resistant to infection, resulting in a suitable chronic model [23]. In recent years, the use of C57BL/6 mice in vaccine studies has been preferred because this mouse strain is considered to mimic chronic human illness and long-term latent infection. Therefore, in this report we wanted to establish the ability of CLH001 vaccine to protect against infection using the chronic mouse model (C57BL/6) for both glanders and melioidosis.

## Methods

### Ethics statement

The animal studies were carried out in strict accordance with the recommendations in the Guide for the Care and Use of Laboratory Animals of the National Institutes of Health, USA. All animal protocols were reviewed and approved by Institutional Animal Care and Use Committee of the University of Texas Medical Branch (protocol no. 0503014D) and the Animal Care and Use Review Office, Department of the Army.

### Bacterial strains and growth conditions

*B*. *mallei* Δ*tonB* Δ*hcp1* was cultured from a freezer stock and plated on LB agar containing 4% glycerol (LBG) and supplemented with 200 μM FeSO_4_·7H_2_O for 72 h at 37°C. The bacterial colonies were inoculated into 20 mL of LBG broth and incubated for 16 h with agitation at 37°C, as previously described [17]. Wild-type *B*. *mallei* ATCC 23344 and *B*. *pseudomallei* K96243 were streaked on LBG agar plate and grown in 20 mL LBG broth at 37°C for 16 h and 12 h, respectively. All the experiments were performed accordingly to Select Agents regulations.

### Animal studies

Female 6 to 8-week-old C57BL/6 mice were purchased from Jackson Laboratories (Bar Harbor, ME, USA) and maintained in an Animal Biosafety Level 3 (ABSL3). Animals were housed in microisolator cages under pathogen-free conditions with food and water *ad libitum* and maintained on a 12 h light cycle.

### Immunization and challenge study

Anesthetized C57BL/6 mice (n = 10 per group) were inoculated intranasally with a prime and 2 boost regimen of PBS (control) or 1.5 x 10^5^ CFU (50 μl) of *B*. *mallei* CLH001 vaccine at 2-week intervals. Immunized mice were challenged 3 weeks after the last vaccination with 314 CFU of *B*. *mallei* ATCC 23344 (wild-type) via the nose-only aerosol route or 3.24 x 10^4^ CFU (3 LD_50_) via i.n. route. For the cross-protection study, mice were challenged via the nose-only aerosol route with 1.07–1.78 x 10^3^ CFU of *B*. *pseudomallei* K96243 (6.94–11.56 LD_50_) [24]. The infected animals were weighed daily and monitored for survival until the end of the study. The survival curves were generated and analyzed using the Kaplan-Meier method (Graph Pad Prism version 6.0c). The percent of survival was compared between PBS and CLH001 immunization, and the statistically significant differences (*P* < 0.05) were analyzed by a Log-rank (Mantel-Cox) test.

### Bacterial burden determination and histopathologic evaluation

At the end of study on day 21 (*B*. *mallei* challenge) or day 27 (*B*. *pseudomallei* challenge), the organs (lungs, livers and spleens) of surviving mice were randomly subdivided for bacterial enumeration or histopathological evaluation. For *B*. *mallei* aerosol challenge, the organs of 3/4 and 6/8 of surviving mice in PBS and CLH001 vaccination, respectively, were used to determine bacterial burden and the rest of them (1/4 of PBS and 2/8 of CLH001) for histopathology. For i.n. challenge with *B*. *mallei*, organs of all surviving mice from PBS vaccination (n = 2) and 8/10 mice of CLH001 vaccination were enumerated for bacterial burden while 2 of them were used to evaluate histopathology. In the cross-protection study with *B*. *pseudomallei* aerosol challenge, 6 of 9 surviving mice in CLH001 vaccination group, organs were diluted and plated for bacterial enumeration and 3 of 9 were chosen for histopathological evaluation. The harvested organs were homogenized in 1 mL of PBS using tissue grinders (Covidien, Mansfield, MA). The homogenized tissues were serially diluted and plated on LBG agar using standard plate counts. All of the remaining homogenized tissue were plated on 150 mm x 15 mm LBG agar plate (Fisher). The organs were collected and placed in 10% formalin, paraffin-embedded, and stained with hematoxylin and eosin (H&E) for histopathology. The stained tissue sections were examined in a blind-manner by a pathologist.

### Detection of *B*. *mallei*-specific antibodies

Baseline and post vaccination sera from PBS-treated and CLH001-immunized mice were collected 5 days before prime and 14 days after the second boost. Whole blood was collected via retro-orbital bleeding and stored in microvette tubes without anti-coagulant. The serum was separated by centrifugation and stored at -80°C. The *B*. *mallei*-specific total IgG, IgG1 and IgG2a titers were determined by indirect ELISA. Briefly, the microplate (Costar, Cambridge, MA) was coated at 4°C overnight with 10 μg/ml of irradiated *B*. *mallei* ATCC 23344 in 1x Dulbecco’s Phosphate-Buffered Saline (DPBS) (Corning). Wells were washed twice with washing buffer (0.05% Tween-20 in 1x DPBS) and then blocked with blocking buffer (0.1% Tween-20, 1% BSA, 1x DPBS) at room temperature for 2 h. The blocked wells were washed twice before adding sample diluent. The sera were added to each top dilution well in triplicate, and two-fold dilutions were performed following incubation at room temperature for 2 h. Diluted goat anti-mouse total IgG, IgG1 or IgG2a antibody (1:5,000) (Southern Biotech) was added into each well and then incubated for 2 h. Plates were washed four times prior adding tetramethylbenzidine (TMB) substrate solution (Invitrogen). Stop solution (2N H_2_SO_4_) was added to stop the reaction, and the samples were immediately read at 450 and 570 nm using a microplate reader (Biotek). The results were reported as the reciprocal of the highest titer giving an optical density (O.D.) reading of at least mean + 2SD of greater than the baseline sera. All assays were performed in triplicate, and results were shown as the mean reciprocal endpoint titer. The Mann-Whitney test was used to analyze a significant difference (*P* < 0.05) in *B*. *mallei*-specific antibody levels between the PBS- and CLH001-immunized mice.

### Examination of cellular immune response

Mice were vaccinated with PBS or CLH001 as described above. Spleens were collected 21 days after the second vaccination. Splenocytes were isolated and seeded at 5 x 10^6^ cells/well in 24 well-plates. The splenocytes were re-stimulated with BSA (negative control), 1 x 10^7^ CFU/well of heat-killed *B*. *mallei* ATCC 23344 or *B*. *pseudomallei* K96243 whole cell lysate (WCL). Plates were incubated at 37°C in 5% CO_2_ for 72 h. Cell culture supernatants were collected, and the concentrations of cytokines (IFN-γ and IL-17A) were measured using ELISA kits purchased from Invitrogen. The data were analyzed using non-parametric one-way ANOVA followed by a Dunn’s test for group comparisons.

### Depletion of CD4^+^ and CD8^+^ T cells after CLH001 immunization

Female 6-to-8-week-old C57BL/6 mice (5 mice/group) were vaccinated using a prime and two-boost regimen with PBS or CLH001 as described above. The depletion of CD4^+^ and CD8^+^ T cells was performed using a previously validated method [19]. Briefly, mice were injected intraperitoneally (i.p.) with 500 μg (the first depletion) and 250 μg (the second to sixth depletion) of IgG2b isotype control (LTF-2), rat anti–mouse CD4 (GK1.5) or rat anti–mouse CD8α (YTS 169.4) (purchased from BioXcell), two weeks after the second boost. The depletion was performed 3 days before (-3), on the day of infection (0) with 4.94 x 10^4^ CFU (~4 LD_50_) of *B*. *mallei* ATCC 23344 via i.n. route and maintained by further administration every 3 days post-infection. Mice were monitored for 16 days, then organs harvested, homogenized and plated to evaluate bacterial burden. A significant difference (*P* < 0.05) of percent survival were determined using a log rank (Kaplan-Meier) test. Bacterial burden in lung, liver, and spleen was determined on day 16 (LOD < 10 CFU/organ). The difference of CFU/organ from each mouse was compared using One-Way ANOVA followed by a Tukey’s test. Further, the peripheral blood (retro-orbital), lungs, and spleens were collected from a separate but matched group of mice (2 mice/group) to allow assessment of the depletion protocol over the experimental time-period. The efficiency of depletion was confirmed by flow cytometry analysis 24 h after staining [19]. Flow cytometry analysis was performed using a BD Fortessa LSR II flow cytometer, and results were analyzed using FCS Express 6 (Glendale, CA, USA).

## Results

### *B*. *mallei* CLH001 immunization and *B*. *mallei* ATCC 23344 wild-type challenge

**Fig 1:**
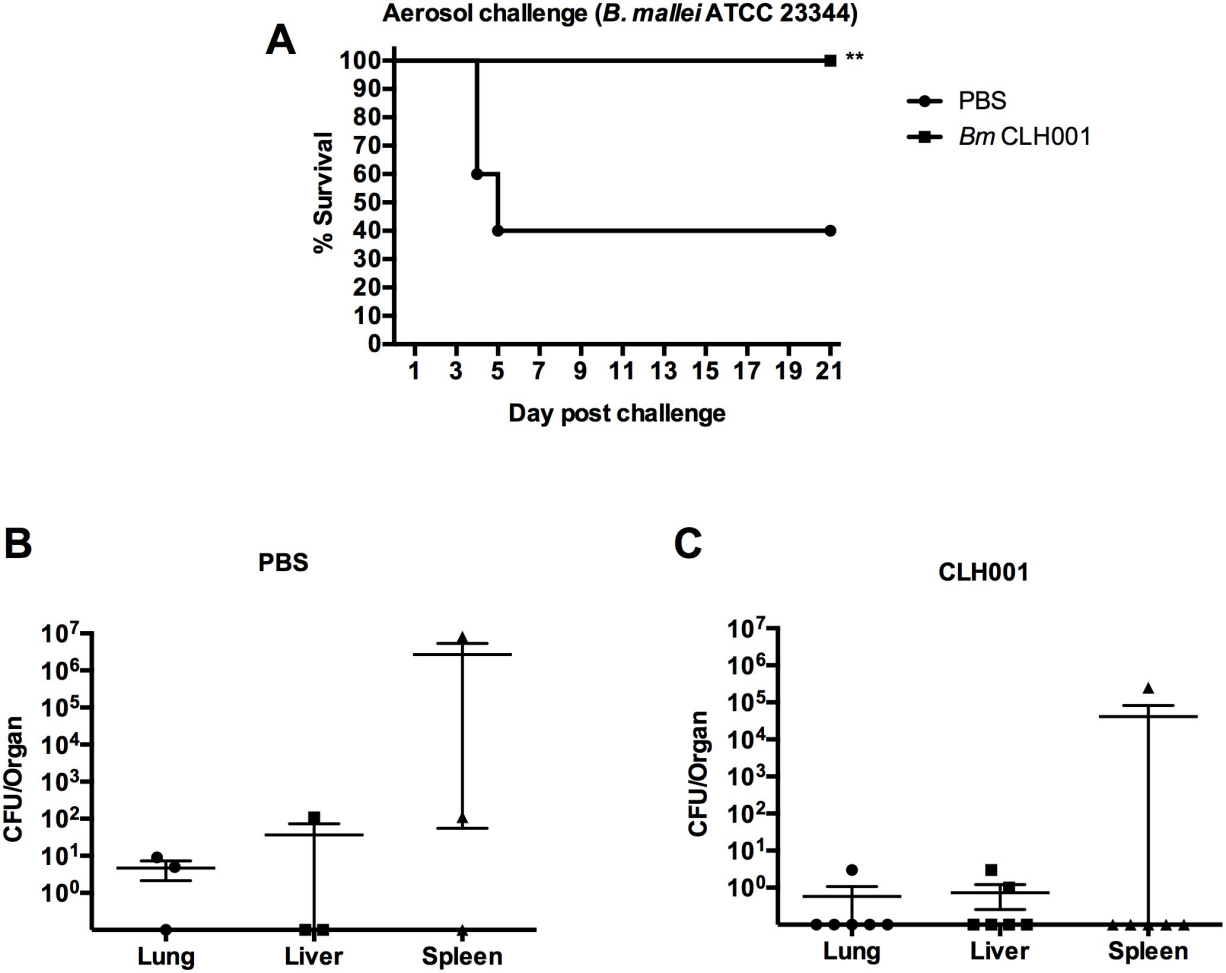
CLH001 vaccine provides 100% protection against aerosol challenge with wild-type *B*. *mallei*. (A) C57BL/6 mice (n = 10 per group) were primed and boosted i.n. with PBS (solid circle) or 1.5 x 10^5^ CFU of CLH001 (solid square). Three weeks after the last boost, mice were challenged via the aerosol route with 314 CFU of *B*. *mallei* ATCC 23344. Survival was analyzed using a log rank (Kaplan-Meier) test (**, *P* < 0.01). (B and C) The organs of surviving mice from PBS and CLH001 vaccination groups were plated to determine bacterial burden.

C57BL/6 mice were immunized with three doses of PBS or CLH001 vaccine at two-week intervals, and then challenged via the aerosol or i.n. route with *B*. *mallei* ATCC 23344 on day 21 after the last boost. For the aerosol challenge experiment, the PBS mice succumbed to infection on day 4 and showed 40% (4/10 mice) survival on day 21 post challenge. In contrast, CLH001-immunized mice showed 100% (8/8 mice) survival without any sign of illness and continued gaining weight until the end of the study (Fig 1A). The survival study of immunized mice after challenge with *B*. *mallei* wild-type strain via i.n. showed the PBS mice succumbing to infection starting on day 3 and showed 20% survival (2/10 mice) on day 21 post infection while mice receiving CLH001 vaccine showed 100% survival (10/10) without any sign of illness during the period of the study (S1 Fig, panel A).

### Vaccination with CLH001 provides cross-protective immunity to *B*. *pseudomallei* aerosol challenge

**Fig 2:**
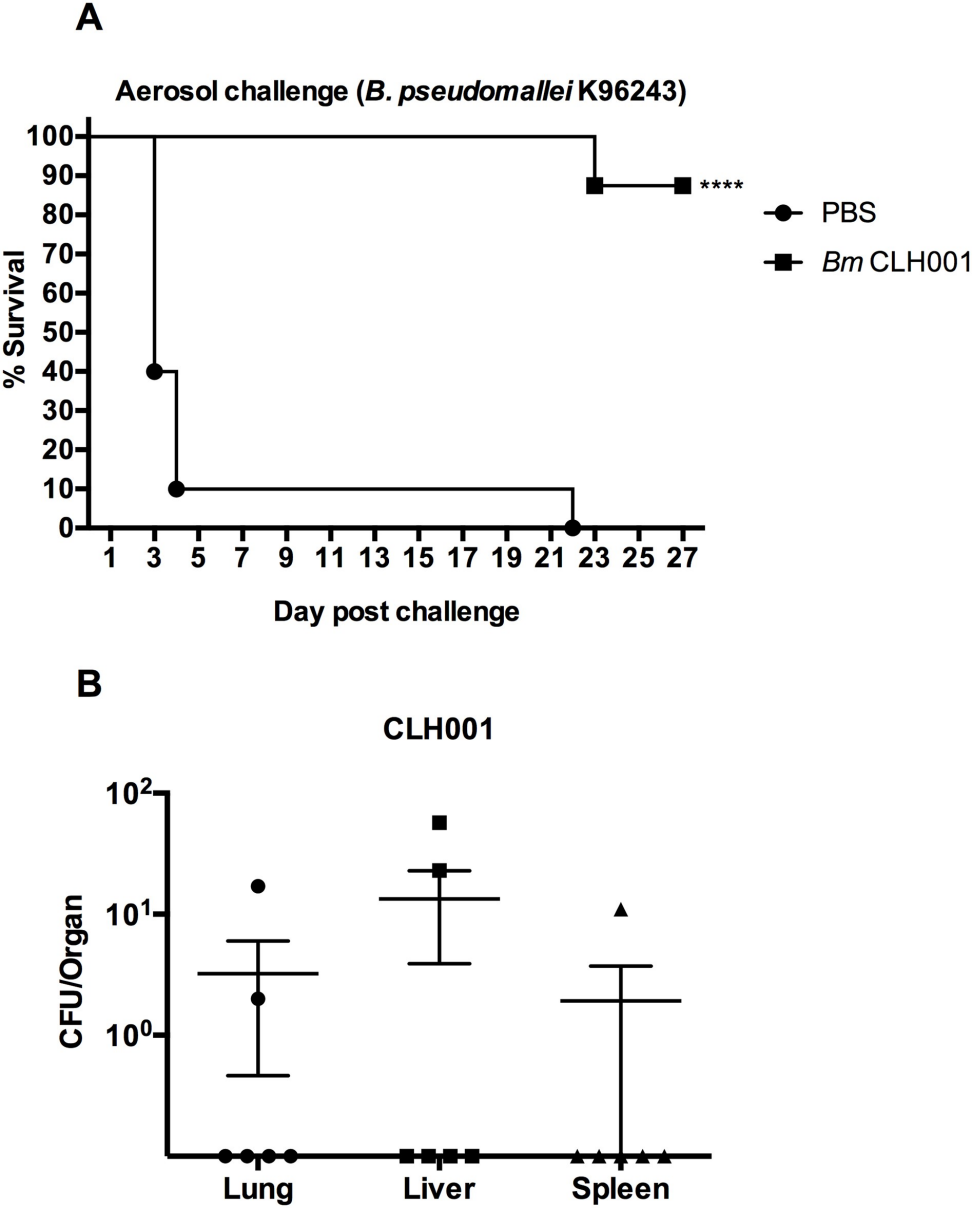
CLH001 vaccine provides increased protection against aerosolized wild-type *B*. *pseudomallei* and low bacterial burden in organs. (A) C57BL/6 mice (PBS; n = 10, CLH001; n = 8) were primed and boosted i.n. with PBS (solid circle) or 1.5 x 10^5^ CFU of CLH001 (solid square). Three weeks after the last boost, mice were challenged with 6.94–11.56 LD_50_ of *B*. *pseudomallei* K96243 via the aerosol route. Survival was analyzed using a log rank (Kaplan-Meier) test (****, *P* < 0.0001). (B) Bacterial burden in lungs, livers and spleens of surviving mice post-challenge was calculated.

For the cross-protection study, CLH001-immunized C57BL/6 mice were challenged via the aerosol route with *B*. *pseudomallei* K96243 on day 21 after the last boost. The CLH001-vaccinated mice showed 100% (8/8) survival without signs of illness or weight loss until day 21 and then dropping to 87.5% (7/8) survival (1 mouse showed neurological signs), which were maintained until day 27 post challenge, while the PBS mice started succumbing to infection on day 3 post-challenge and all the mice died by day 22 (Fig 2A).

### Bacterial organ burdens

The organs of mice after *B*. *mallei* wild-type challenge were collected 21 days post infection (dpi). The lung, liver, and spleen of surviving animals were processed. The aerosol challenge study showed bacterial infection in 2 of 3 surviving mice from the PBS-vaccinated group (mouse #1: 9 CFU/lung, 0 CFU/liver and 1.10 x 10^2^ CFU/spleen, mouse #2: 5 CFU/lung, 1.09 x 10^2^ CFU/liver and 8.08 x 10^6^ CFU/spleen); however, one of the mice had no bacterial detection in lung, liver or spleen, suggesting that the mouse might have cleared the aerosolized bacteria or the challenge strain might disseminate to other organs (Fig 1B). From the surviving mice that received the CLH001 vaccine, there was no detectable bacteria in any of the 3 organs in 3 of the 6 mice, lower bacterial infection in 2/6 mice and one high bacterial burden in the spleen (2.48 x 10^5^ CFU/spleen) of another mouse (Fig 1C).

For the i.n. challenge study, 2 PBS-vaccinated surviving mice displayed high bacterial burden in spleen (2.11 x 10^5^ and 9.4 x 10^6^ CFU/organ), while surviving mice of the CLH001-vaccinated group showed no detectable bacterial burden in all 3 organs of the 7/8 surviving mice (87.5%), but one mouse showed bacterial infection in lung (2.73 x 10^6^ CFU), liver (3.44 x 10^4^ CFU) and spleen (9.3 x 10^3^ CFU) (S1 Fig, panel B-C).

The cross-protection study of the CLH001 vaccine against *B*. *pseudomallei* wild-type aerosol challenge showed low bacterial infection (<100 CFU/organ) in 3 of 6 mice and no bacterial infection in the 3 organs of the other 3/6 mice (Fig 2B). The result from determination of bacterial burden in organs of surviving mice indicated that immunization with CLH001 significantly reduced the bacterial levels of both *B*. *mallei* and *B*. *pseudomallei* infections.

### Detection of *B*. *mallei*-specific antibodies

**Fig 3:**
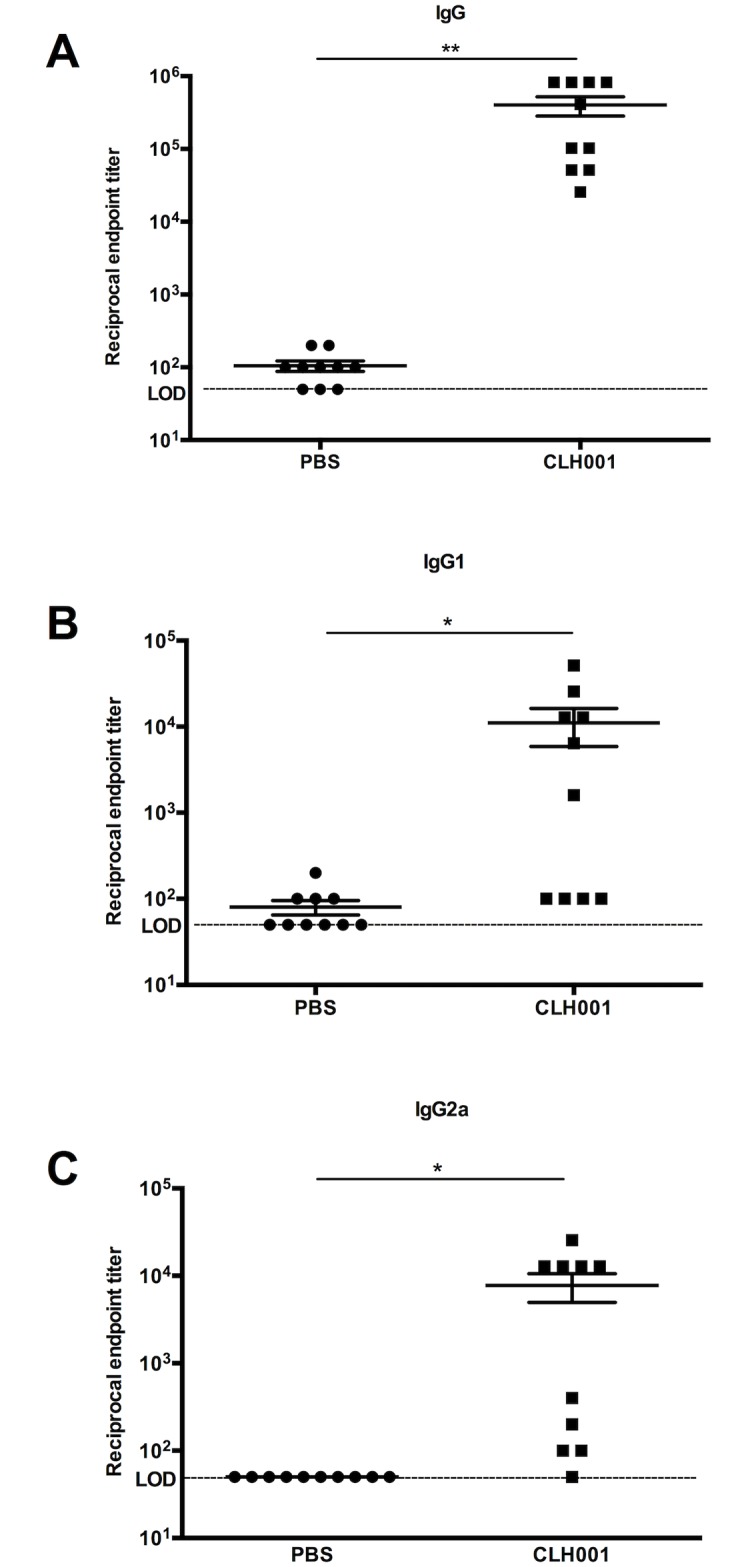
*B*. *mallei*-specific antibody responses after CLH001 vaccination. Serum samples from mice (n = 10 per group) immunized with PBS or CLH001 were collected two weeks after the last boost. The total serum IgG (A), IgG1 (B) and IgG2a (C) antibodies were determined by an indirect ELISA using *B*. *mallei* whole cell lysates. The antibody levels are presented as the reciprocal of the highest titer giving an optical density (OD) reading of at least the mean + 2SD of baseline sera. The significant differences between PBS- and CLH001-vaccinated groups were analyzed using the Mann-Whitney test (*, *P* < 0.05, **, *P* < 0.01). The limit of detection (LOD) is 50 (horizontal dotted line). Bars represent means ± standard errors of the mean (SEM).

Serum was collected from PBS-treated or CLH001-vaccinated mice at 14 days after the final vaccination boost. The serum was used to determine the endpoint titer of *B*. *mallei*-specific total IgG, IgG1 and IgG2a using an indirect ELISA. Mice receiving the CLH001 vaccine showed significantly higher endpoint titers of IgG (mean = 401,920), IgG1 (mean = 11,080), and IgG2a (mean = 7,765) when compared to mice receiving PBS (mean of IgG = 105, IgG1 = 80 and IgG2a = 80) (Fig 3). The mean value was used to calculate the IgG1/IgG2a ratio as 1.43, indicating that a predominant type 2 (Th2) antibody response in mice following vaccination with CLH001.

### Cellular immune responses

**Fig 4:**
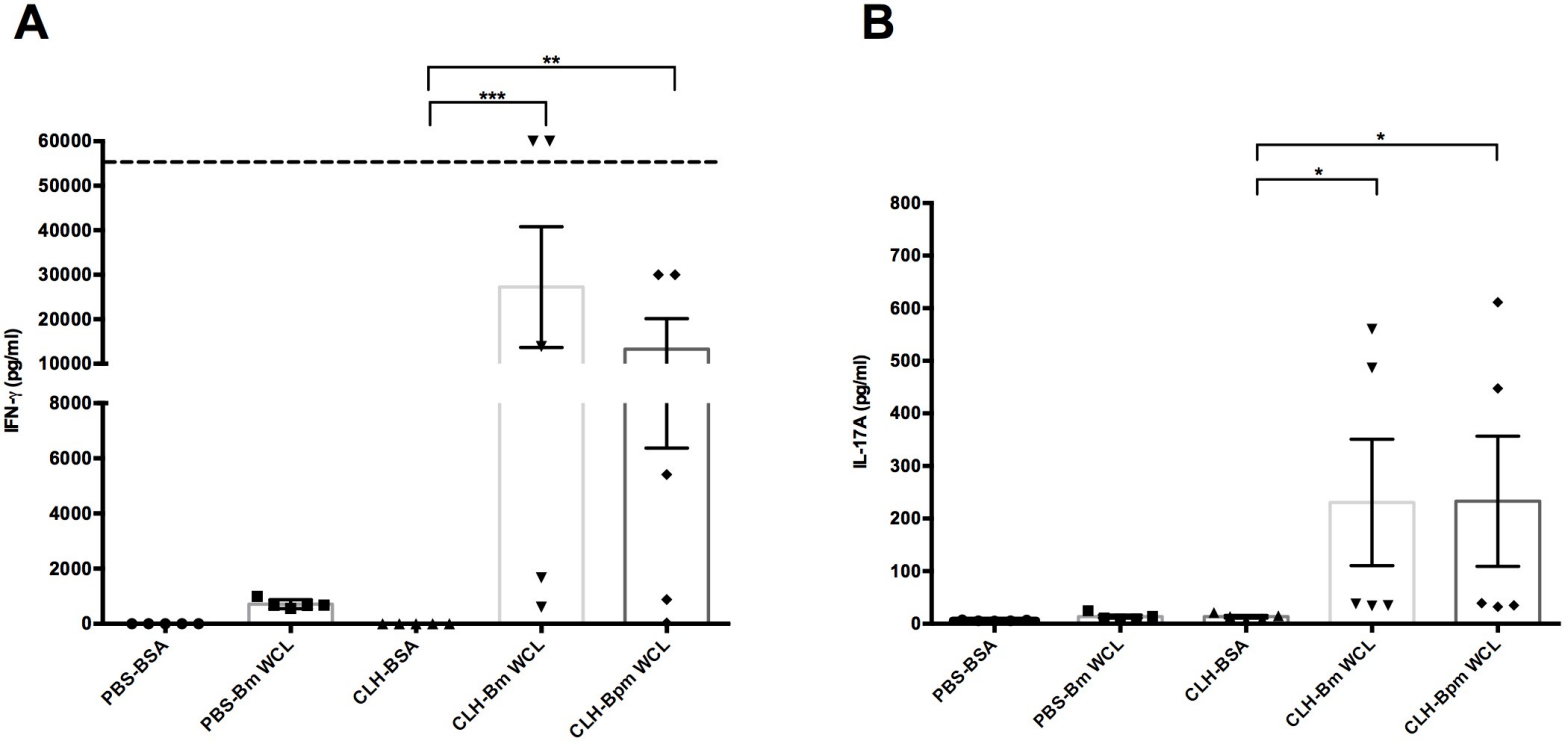
Vaccination with CLH001 induces cellular immune responses. Splenocytes were isolated from PBS- or CLH001-vaccinated mice (n = 5 mice per group) on day 21 after the last vaccination. Isolated splenocytes were re-stimulated with either BSA (negative control), heat-killed *B*. *mallei* ATCC 23344 or *B*. *pseudomallei* K96243 whole cell lysates (WCL) for 3 days. The antigen specific IFN-γ (A) and IL-17A (B) levels in cell supernatants were measured by ELISA. Data were analyzed using non-parametric one-way ANOVA followed by Dunn’s test (n.s., not significant; *, *P* < 0.05; **, *P* < 0.01; ***, *P* < 0.001). The horizontal broken line represents the maximum limit of detection.

The cellular immune response in CLH001 vaccine-induced protection was examined on day 21 after the last vaccination. The spleens of PBS and CLH001-vaccinated mice were collected and the splenocytes isolated, seeded, and re-stimulated with either BSA (negative control), heat-killed *B*. *mallei* ATCC 23344 WCL, or heat-killed *B*. *pseudomallei* K96243 WCL for 72 h. The splenocytes from mice vaccinated with CLH001 showed significantly higher IFN-γ and IL-17A cytokines after stimulation with *B*. *mallei* and *B*. *pseudomallei* WCL as compared to BSA (negative control) (Fig 4A and 4B). There was no significant production of IFN-γ or IL-17A in splenocytes of PBS mock-vaccinated animals following exposure with *B*. *mallei* and *B*. *pseudomallei* WCL antigens. The IL-17A response was significantly higher in CLH001-vaccinated splenocytes compared to PBS splenocytes. These results demonstrated that the CLH001 vaccine induced an antigen specific cellular response by production of effector cytokines characteristic of Th1 (IFN-γ) and Th17 (IL-17A) immune responses.

### Depletion of CD4^+^ and CD8^+^ T cells after CLH001 immunization

**Fig 5:**
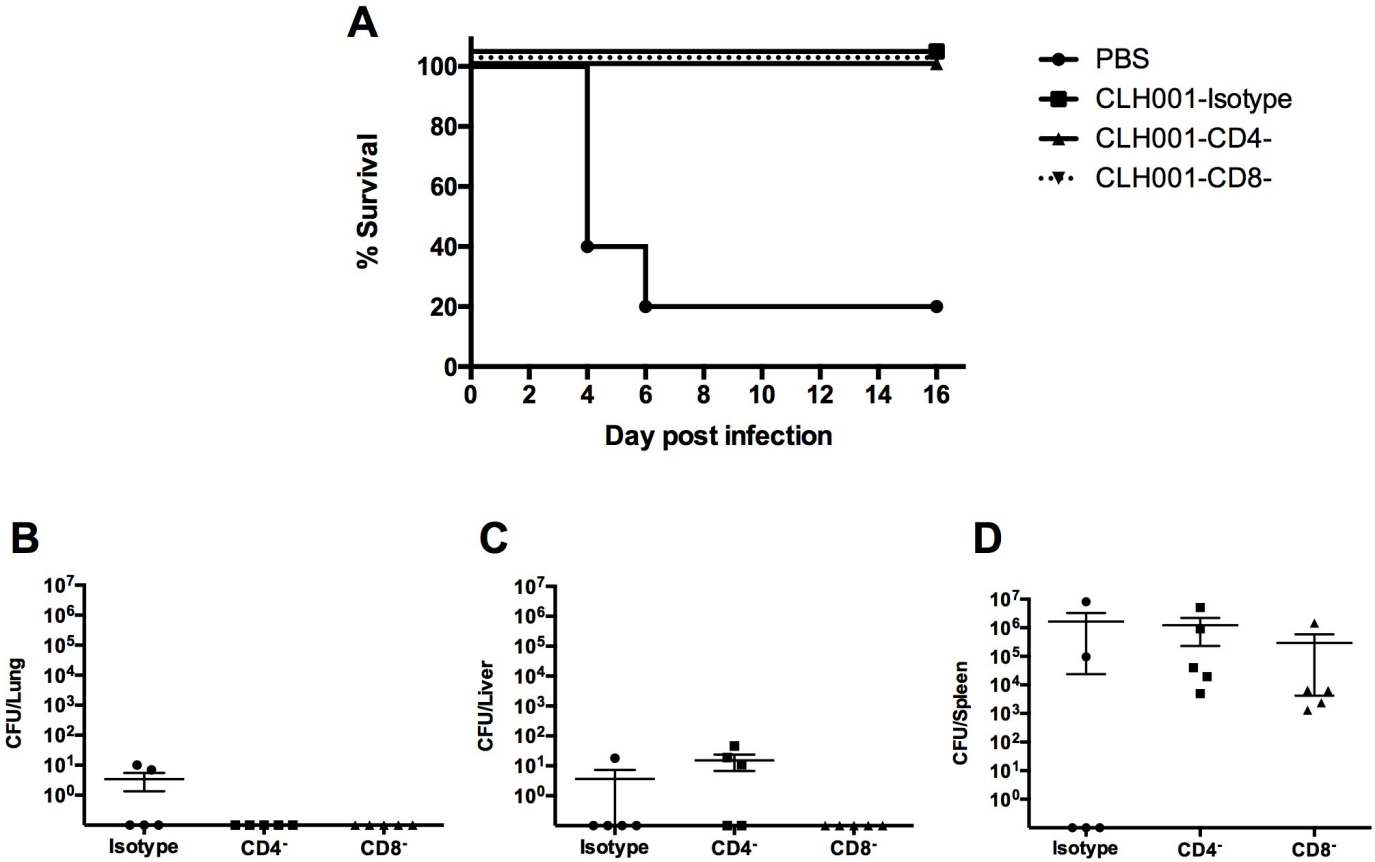
The depletion of T cell populations shows no difference in protection or bacterial burden in CLH001-vaccinated mice. (A) C57BL/6 mice (n = 5 per group) were immunized with PBS or CLH001 and then treated with rat IgG2b isotype control, anti-mouse CD4 or anti-mouse CD8α at day 14 after last boost. The depletion was performed 3 days prior challenge, on the day of challenge (with 4 LD_50_ of *B*. *mallei* ATCC 23344 via i.n. route) and maintained every 3 days post challenge. Mice were monitored for survival and differences were determined using a log rank (Kaplan-Meier) test. The bacterial burden in lungs (B), livers (C) and spleens (D) were enumerated on day 16 post-challenge (LOD < 10 CFU/organ). The significant difference of CFU/organ from each treatment group was compared using one-way ANOVA followed by Turkey’s test. Bars represent means ± standard errors of the mean (SEM).

CLH001-immunized mice were depleted of individual T cell populations prior to challenge with *B*. *mallei* ATCC 23344 to determine the protective role of either CD4^+^ or CD8^+^ T cells. The depletion efficiencies were confirmed by flow cytometric analysis of CD4^+^ and CD8^+^ T cell populations in an age and gender-matched group of non-infected mice and reported separately in a similar study [19]. The results demonstrated that the depletion protocol resulted in 99.78–100% depletion of CD4^+^ and 98.90–99.50% of CD8^+^ T cells in the lung, spleen, and peripheral blood [19]. The percent survival of animals was used to evaluate the effect of T cell depletion prior to challenge. Mice receiving PBS succumbed to infection at 4 dpi, while CLH001-immunized mice replete with T cell (isotype control) as well as CLH001-immunized mice depleted of CD4^+^ and CLH001-CD8^+^ T cells showed 100% survival at 16 dpi (Fig 5A). The lungs, livers and spleens of surviving mice at the end of study were collected and CFU/organ enumerated to determine the effect of T cell depletion on controlling bacterial burden. The results showed low bacterial burden in lungs and livers of all treatment groups (Fig 5B and 5C). Spleens of all groups showed high level of bacterial infection (Fig 5D). However, there was no significant difference in bacterial concentrations in lungs, livers, and spleens among isotype control, CD4^+^ and CD8^+^ depleted T cells. These results indicate that CD4^+^ and CD8^+^ T cells do not appear to contribute significantly to CLH001 vaccine-induced protection.

### Histopathologic evaluation

**Fig 6:**
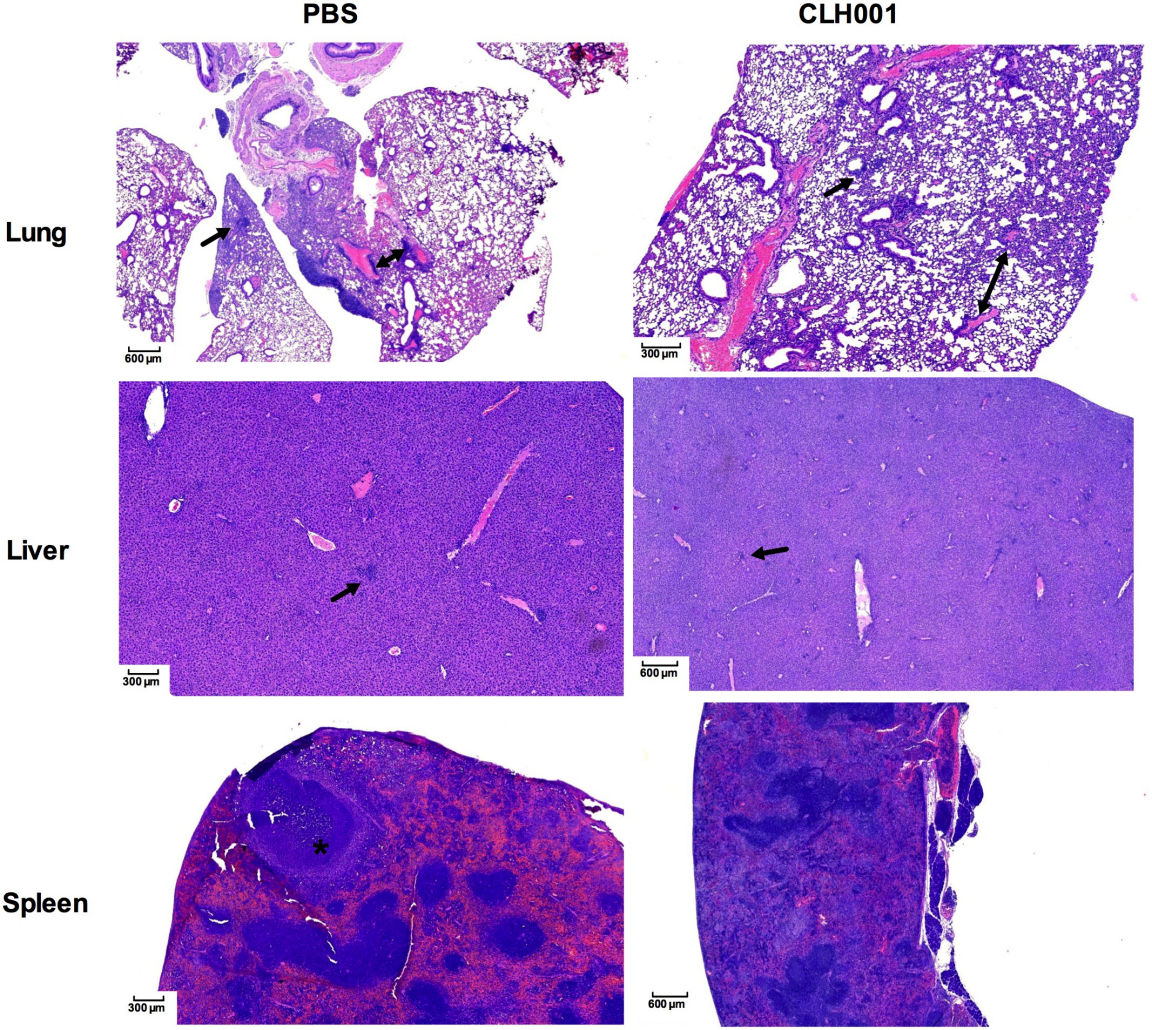
Histopathologic analysis of mice immunized with PBS or CLH001 and post-challenge with *B*. *mallei* ATCC 23344. Lung, liver and spleen of surviving mice were harvested at the end of the study (21 dpi). The organs were fixed, processed and stained with H&E. The figures represent images captured at of 2X (scale bar = 600 μm) and 4X (scale bar = 300 μm) magnification. Arrows represent inflammatory foci in lung and liver. Double arrow represents perivascular infiltration of inflammatory cells in lung and an asterisk shows abscess in spleen.

Lungs, livers, and spleens of surviving mice at the endpoint of the study were collected, processed for H&E staining, and blindly examined by a pathologist. The organ sections of mice exposed via aerosol challenge to *B*. *mallei* ATCC 23344 are shown in Fig 6. The lung of a surviving mouse (n = 1) from the PBS group showed peribronchial and perivascular plasma cell-rich infiltrates, interstitial pneumonia consisting of infiltration of alveolar septa with macrophages and lymphocytes, and subpleural lymphoid nodules (6.5 ± 2.1 foci per ten 40X magnification fields). Liver showed numerous lobular foci of cellular infiltrates consisting of polymorphonuclear leukocytes, lymphocytes, and macrophages with rare apoptotic hepatocytes (5.5 ± 0.7 foci per ten 40X magnification fields). Spleen showed abscesses, abundant karyorrhectic nuclear fragments and polymorphonuclear leukocytes in red pulp (1.0 ± 1.4 foci per ten 40X magnification fields). Interestingly, the lungs of CLH001-vaccinated mice (n = 2) showed less extensive peribronchial and perivascular infiltrates of lymphocytes, which contained more numerous plasma cells and macrophages than PBS-vaccinated mice but less interstitial pneumonia than the PBS-vaccinated mice. The number of inflammatory foci in the lung of CLH001-vaccinated mice was 5.5 ± 0.7 foci per ten 40X magnification fields. The livers contained few inflammatory foci (4.0 ± 0.0 foci per ten 40X magnification fields) containing lymphocytes, polymorphonuclear leukocytes, and macrophages, most of which are smaller than those in the PBS-vaccinated mice, and apoptotic hepatocytes were observed in some inflammatory foci. Lymphoid hyperplasia with large periarteriolar lymphocytic sheaths, many of which contained germinal centers were observed in spleen. The few numbers of inflammatory foci in spleen of CLH001-immunized mice was estimated as 0.5 ± 0.7 foci per ten 40X magnification fields.

The stained tissues from surviving mice after i.n. challenge with *B*. *mallei* wild-type indicated that one large subpleural macrophage-rich infiltrate also containing many polymorphonuclear leukocytes, plasma cells, and lymphocytes was observed in lung of a mouse vaccinated with CLH001. The number of foci in lung was 2.5 ± 0.7 foci per ten 40X magnification fields. The number of foci in liver was 2.5 ± 0.7 foci per ten 40X magnification fields. The spleen of CLH001-vaccinated mice contained no pathologic lesions (S2 Fig). These observations reflect an effective immune response elicited by the vaccine.

**Fig 7:**
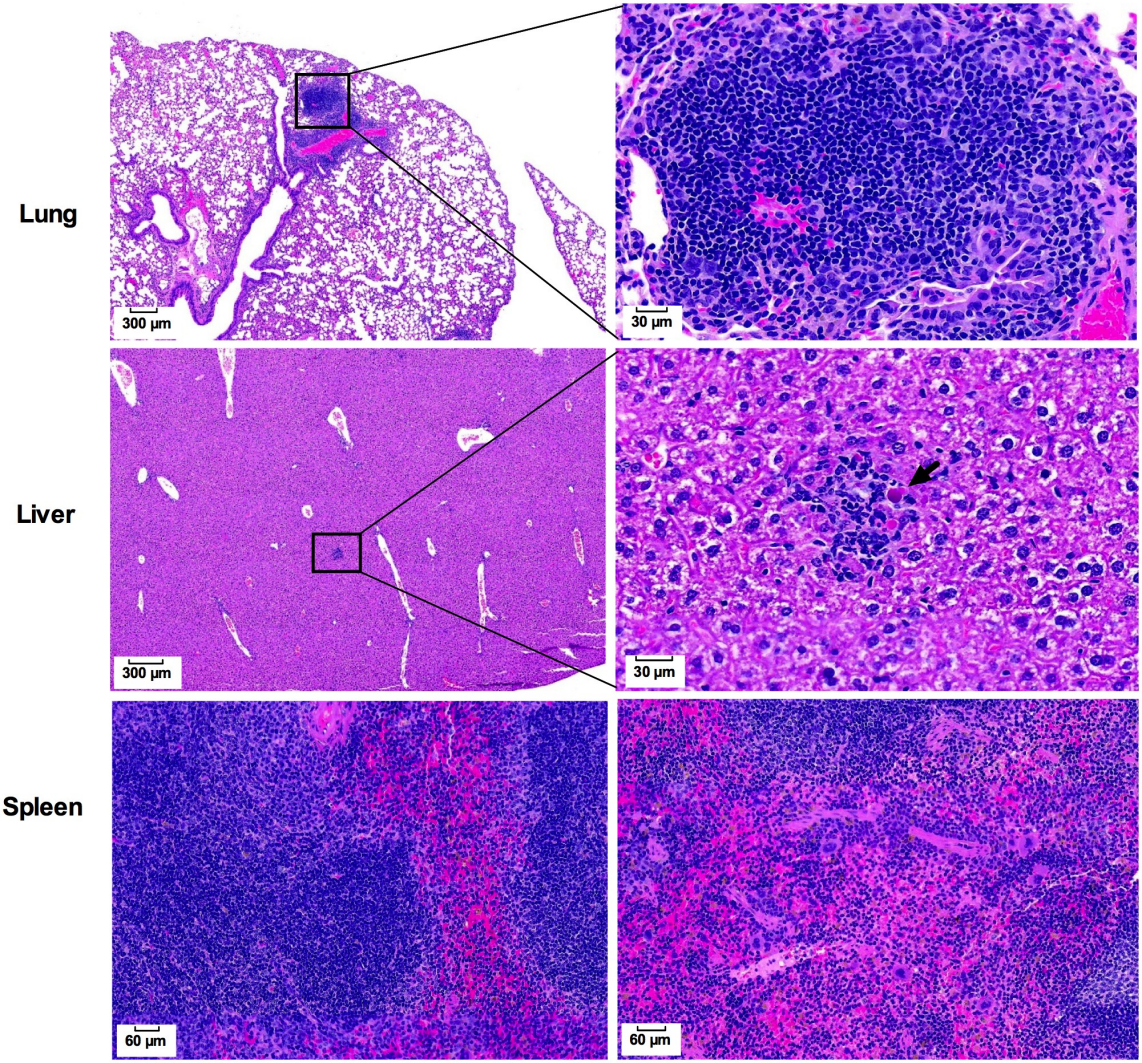
Histopathology representing organs of CLH001-immunized mice post-challenge with *B*. *pseudomallei* K96243. Organs of surviving mice were harvested at the end of study (27 dpi). The tissues were fixed, processed and stained with H&E. The figures represent images captured at 4X (scale bar = 300 μm), 20X (scale bar = 60 μm) and 40X (scale bar = 30 μm) magnification. The lymphocyte-rich inflammatory foci in lung and mixed lymphocyte, macrophage, and polynuclear leukocyte infiltrate in liver of CLH001-vaccinated mice are represented in squares, and magnifications are shown in the right panels. An arrow represents apoptotic hepatocyte in liver of CLH001- vaccinated mice.

In the cross-protection study, the organs of CLH001-vaccinated mice exposed by the aerosol route to *B*. *pseudomallei* K96243 were also evaluated for histopathology (n = 3) and the representative images are shown in Fig 7. The lung showed lymphocyte-rich inflammation around the bronchoalveolar tree (3.7 ± 1.4 foci per ten 40X magnification fields). Liver showed multiple focal lobular mixed inflammatory infiltrates (lymphocytes, macrophages and granulocytes) with occasional association with an apoptotic hepatocyte, and the number of inflammatory foci was 2.8 ± 1.9 foci per ten 40X magnification fields. Prominent periarteriolar lymphoid sheaths and abundant hematopoiesis were observed in spleen of CLH001-immunized mice.

Overall, reduction in pathological changes was found in CLH001-vaccinated mice compared to PBS mice after exposure to *B*. *mallei* challenge. Moreover, the histopathologic evaluation indicated that minimal to moderate changes in organ architecture occurred in CLH001-vaccinated mice after challenge with the *B*. *mallei* and *B*. *pseudomallei* wild-type strains.

## Discussion

We have previously demonstrated that an attenuated, safe and effective *B*. *mallei* CLH001 vaccine was able to protect BALB/c mice against *B*. *mallei* challenge [17]. In the current study, we evaluated *B*. *mallei* CLH001 vaccine against glanders and melioidosis disease in C57BL/6 mice, which are more resistant to acute infection. We found that CLH001 conferred full protection from death against inhalational glanders and demonstrated a high level of cross-protection against inhalational melioidosis.

To test and develop effective and safe glanders and melioidosis vaccines, BALB/c and C57BL/6 mouse strains are mostly used based on the different disease outcomes observed [20,21,22]. In the past few years, BALB/c mice were used to perform virulence studies and vaccine development for both melioidosis and glanders [20,23] because they developed an acute infection as a susceptible host. In contrast, C57BL/6 mice are more resistant to infection and are considered a more suitable model to study chronic melioidosis infection [25]. Because our prior study was performed using the i.n. route as a challenge route, we decided to expand our current study and do a comparative analysis of i.n. and aerosol challenges in vaccinated C57BL/6 mice. Prime and two boosts vaccination schemes with CLH001 via the i.n. route provided 87.5% survival of BALB/c mice after challenged with high dose (140 LD_50_) of *B*. *mallei* ATCC 23344 [17] whereas the same dose regimen provides 100% survival in C57BL/6 mice after (3 LD_50_) challenge (S1 Fig, panel A). Our prior study did not detect bacteria in lungs of BALB/c mice, but bacterial counts were found in livers and mostly in spleens. In our current comparative study, C57BL/6 mice showed fewer bacterial numbers in lungs, livers and spleens (S1 Fig, panel C). Interestingly, the CLH001 vaccine cross-protection study against *B*. *pseudomallei* K962343 infection in BALB/c and C57BL/6 mice displayed different outcomes. Using the same vaccination scheme, CLH001-vaccinated BALB/c mice showed partial cross-protection (12.5% survival) with *B*. *pseudomallei* K962343 challenge. In contrast, 87.5% survival was observed in CLH001-vaccinated C57BL/6 mice after challenged with aerosolized *B*. *pseudomallei* K962343. In comparison with previous studies, the bacterial numbers in surviving animals were much lower in C57BL/6 mice than BALB/c mice. These results suggest a failure of the host to control disseminated bacteria while using the BALB/c mouse model, resulting in an acute form of the infection. Overall, we have now shown that the CLH001 live attenuated vaccine strain has protection potential against *B*. *mallei* infections (i.n. or aerosol) and demonstrates significant cross-protection against *B*. *pseudomallei* infection.

Due to the intracellular lifestyle and virulence profiles of *B*. *mallei* and *B*. *pseudomallei*, prior studies have proposed that a cell-mediated immune response generated by an ideal vaccine should be a critical requirement to eliminate bacteria from infected host cells and prevent latent infections [15,26,27,28,29,30]. However, our recent findings characterizing the humoral and cellular immune responses in C57BL/6 mice following *B*. *pseudomallei* PBK001 vaccination indicated that the antibody responses played a critical role in protection, whereas bacteria-specific CD4^+^ and CD8^+^ T cells responses played a minor role [19]. We now show consistent and similar results with our CLH001 vaccine study. The CLH001 vaccine elicited a robust humoral immune response by production of *B*. *mallei*-specific total IgG, as well as IgG1 and IgG2a isotypes. Moreover, re-stimulation of splenocytes from CLH001-vaccinated mice with *B*. *mallei* ATCC 23344 and *B*. *pseudomallei* K96243 WCL indicated that the cellular immune response induced IFN-γ and IL-17A production. It is possible that CD4^+^ and CD8^+^ T cells responses may be mostly dispensable at the time of challenge but may be critical at the time of vaccination, when B cell memory development and isotype class switching occur. The strong IgG response is consistent with development of a strong T helper response during the priming stage. Further, our data suggest that although cell mediated responses may not play a significant role at the time of challenge, CD4^+^ T cell helper response required to induce optimum humoral immunity was generated by CLH001 vaccination. Long term, it will be important to further define the role T cells during priming as opposed to their direct protective effector function at the time of challenge.

Regarding antibodies playing critical role in vaccine-mediated protection against *Burkholderia* infections, other groups have previously evaluated their role in protection against glanders and melioidosis. For example, passive transfer of immune serum from mice immunized with *B*. *pseudomallei purM* (Bp82) and *B*. *mallei batA* live attenuated strains conferred 38% survival (against heterologous *B*. *pseudomallei* K96243 strain) and 80% survival (against homologous *B*. *mallei* ATCC 23344 strain), respectively [31,32]. Furthermore, immune sera from mice vaccinated with outer membrane vesicles (OMV) obtained from *B*. *pseudomallei* 1026b provided protection to 80% of animals following heterologous challenge with *B*. *pseudomallei* K96243 [33]. Altogether, these studies strongly support the idea that vaccination with CLH001 induced mainly antibodies and to a lesser degree cellular immune response, conferring protection. However, the efficiency of antibody responses following CLH001 vaccination in aspects such as kinetics, quantity and quality needs to be further investigated using passive-transfer of immune serum, opsonophagocytic assays and blockage of intracellular replication in macrophages.

Our prior report also demonstrated that unremarkable pathology was observed in organs of BALB/c mice after received the attenuated CLH001 vaccine strain, resembling organs from control mice [17]. In the current study, we further evaluated the organ histopathology of surviving C57BL/6 vaccinated mice after challenge with wild type *B*. *mallei* or *B*. *pseudomallei*. The results indicated that reduced histopathological changes and number of inflammatory foci were present in CLH001-vaccinated compared to mock immunized mice. The lung of CLH001-vaccinated mice, as the portal of entry of the bacteria delivered via the i.n. (S2 Fig) or aerosol challenge routes (Figs 6 and 7), displayed some histologic changes that were even less in liver and spleen. These discrete histopathological observations suggested that vaccination with CLH001 resulted in the induction of an immune response that restricted the infection and prevented disseminated disease in distant organs. Our observations correlated with the degree of bacterial clearance observed in CLH001-vaccinated mice after exposure to *B*. *mallei* via the i.n. (88% clearance) (S1 Fig, panel C) and aerosol (50% clearance) (Fig 1) routes, or aerosolized *B*. *pseudomallei* (50% clearance) (Fig 2) challenge.

In conclusion, our results strongly demonstrate a major role of the humoral immune responses in the protective capacity of the CLH001 vaccine against lethal infections with *B*. *mallei* and *B*. *pseudomallei*. We have reached an interesting cross-road in vaccine development, because we think it will be interesting to determine whether improving cell-mediated immune responses following CLH001 vaccination, by addition of an adjuvant, will further improve protection, generating an enhanced sterilizing immunity and complete bacterial clearance. Finally, evaluation of CLH001 vaccine against various strains of *Burkholderia* spp. challenge will provide informative evidence that this vaccine is cross-protective and can be advanced in the clinical pipeline against different *Burkholderia* species.

## Supporting information

S1 Fig CLH001 vaccine provides full protection after i.n. challenge with wild type *B*. *mallei*. (A) C57BL/6 mice (n = 10 per group) were primed and boosted i.n. with PBS (solid circle) or 1.5 x 10^5^ CFU of CLH001 (solid square). Three weeks after the last boost, mice were challenged i.n. with 3 LD_50_ (3.24 x 10^4^ CFU) of *B*. *mallei* ATCC 23344. The survival was analyzed using a log rank (Kaplan-Meier) test (***, *P* < 0.001). (B and C) The organs of surviving mice from immunized PBS and CLH001 groups were enumerated for CFU/organ.
(TIFF)

S2 Fig Histopathologic analysis of mice immunized with CLH001 post-i.n. challenge with *B*. *mallei* ATCC 23344. Lung, liver and spleen of surviving mice were collected on 21 dpi. Tissues were fixed, processed and stained with H&E. The figures represent images of 4X (scale bar = 300 μm) magnification. Lung of CLH001-immunized mice exposed to *B*. *mallei* ATCC 23344 via i.n. route (n = 2) showed few substantial peribronchial, perivascular and interstitial cellular infiltrates of lymphocytes, macrophages, plasma cells, and PMNs (arrow). Numerous foci (arrow) of lobular, perivascular, and peribronchial cellular infiltrates containing lymphocytes, macrophages, and polymorphonuclear leukocytes and focal apoptosis were observed in liver. The spleen of CLH001-vaccinated mice contained no pathologic lesions.
(TIFF)
