# Supplementary figures and images for: Evaluation of Burkholderia mallei ΔtonB Δhcp1 (CLH001) as a live attenuated vaccine in murine models of glanders and melioidosis
Source: PLoS Negl Trop Dis. 2019 Jul 15;13(7):e0007578. doi: 10.1371/journal.pntd.0007578 (PMC6658008; doi:10.1371/journal.pntd.0007578)

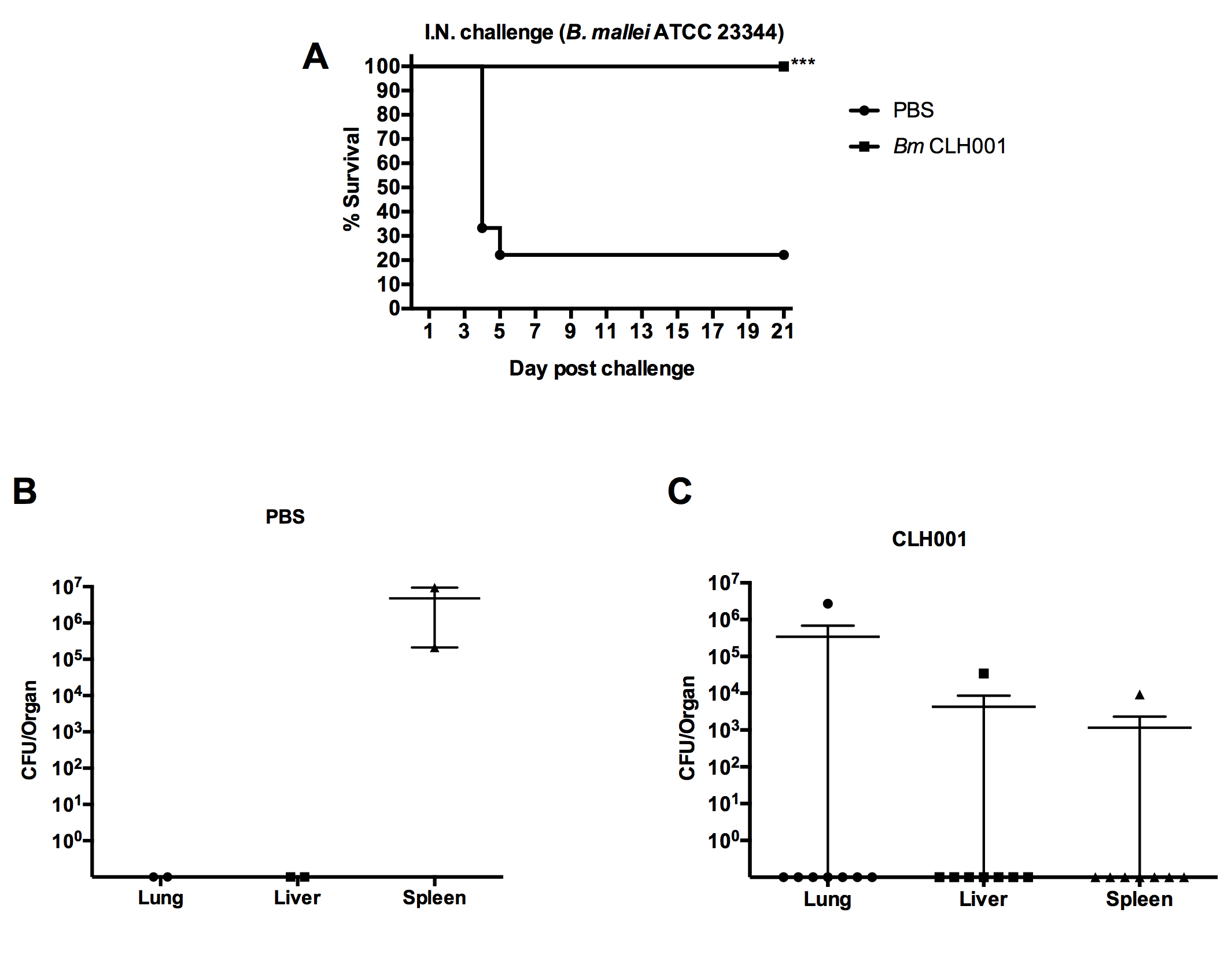

Supplement: S1 Fig — (A) C57BL/6 mice (n = 10 per group) were primed and boosted i.n. with PBS (solid circle) or 1.5 x 10^5^ CFU of CLH001 (solid square). Three weeks after the last boost, mice were challenged i.n. with 3 LD50 (3.24 x 10^4^ CFU) of B. mallei ATCC 23344. The survival was analyzed using a log rank (Kaplan-Meier) test (***, P < 0.001). (B and C) The organs of surviving mice from immunized PBS and CLH001 groups were enumerated for CFU/organ. (TIFF) [file pntd.0007578.s001.tiff]

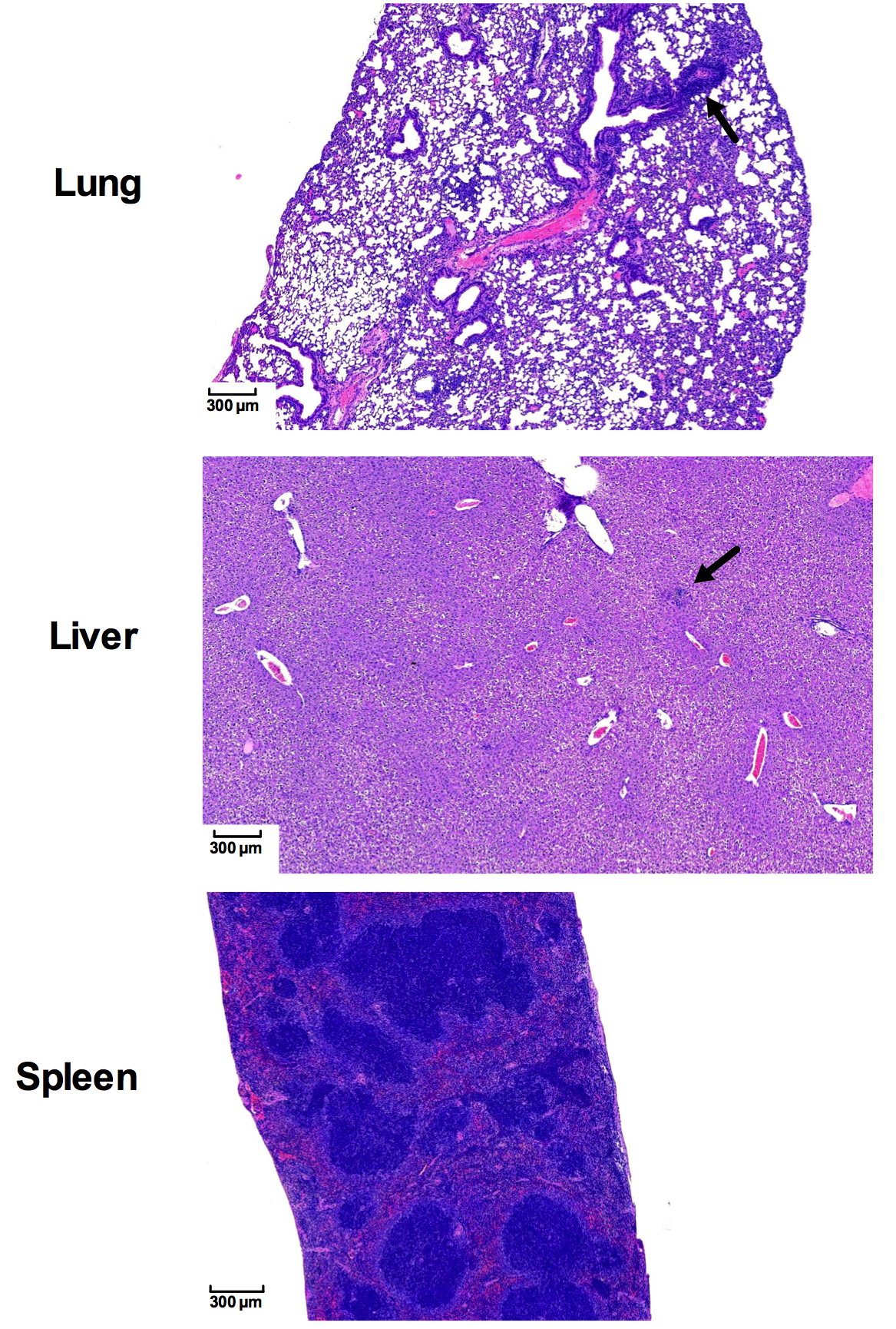

Supplement: S2 Fig — Lung, liver and spleen of surviving mice were collected on 21 dpi. Tissues were fixed, processed and stained with H&E. The figures represent images of 4X (scale bar = 300 μm) magnification. Lung of CLH001-immunized mice exposed to B. mallei ATCC 23344 via i.n. route (n = 2) showed few substantial peribronchial, perivascular and interstitial cellular infiltrates of lymphocytes, macrophages, plasma cells, and PMNs (arrow). Numerous foci (arrow) of lobular, perivascular, and peribronchial cellular infiltrates containing lymphocytes, macrophages, and polymorphonuclear leukocytes and focal apoptosis were observed in liver. The spleen of CLH001-vaccinated mice contained no pathologic lesions. (TIFF) [file pntd.0007578.s002.tiff]
